# National Development Generates National Identities

**DOI:** 10.1371/journal.pone.0146584

**Published:** 2016-02-03

**Authors:** Tea Golob, Matej Makarovič, Jana Suklan

**Affiliations:** School of Advanced Social Studies in Nova Gorica, Nova Gorica, Slovenia; University of Maribor, SLOVENIA

## Abstract

The purpose of the article is to test the relationship between national identities and modernisation. We test the hypotheses that not all forms of identity are equally compatible with modernisation as measured by Human Development Index. The less developed societies are characterised by strong ascribed national identities based on birth, territory and religion, but also by strong voluntarist identities based on civic features selected and/or achieved by an individual. While the former decreases with further modernisation, the latter may either decrease or remain at high levels and coexist with instrumental supranational identifications, typical for the most developed countries. The results, which are also confirmed by multilevel regression models, thus demonstrate that increasing modernisation in terms of development contributes to the shifts from classical, especially ascribed, identities towards instrumental identifications. These findings are particularly relevant in the turbulent times increasingly dominated by the hardly predictable effects of the recent mass migrations.

## Introduction

People may feel the belonging to their societies in various ways, ranging from strong blood kinship relationships, typical for the tribal communities, to the pragmatic, instrumental motives, usually linked to the modern (post) industrial societies. Although nations and national identities are usually seen as a product of modernity [1,2], the ways, in which they are felt and experienced, are not necessarily compatible with the features of a modern society. The purpose of this article is to identify the different or even opposing types of national identity and test their relationship with a developed modern society [3,4]. Based on the existing theory and empirical survey data we intend to distinguish between:

1. ascribed national identities based on birth, territoriality and religion
2. voluntarist national identities based more on choices and achievements
3. instrumental trans- and supranational identifications

We hypothesise that the exclusivist nature of the first contradicts modernity and thus declines with the further social development; the second may be compatible with modernity but there is no elective affinity between the two phenomena; while the third will only increase with the rise of modern development. It will thus be tested, whether modern development also generates different types of national identities.

We intend to measure modernity in quantitative terms using the well-established United Nations measure of Human Development Index (HDI) [5] as a combination of material welfare, health and education. Identity measures, on the other hand, will be based on the indices developed from the available survey data from the International Social Survey Programme (ISSP) [6,7] and the Eurobarometer [8].

## National Identity and Modernity

National identities are clearly related to modernity, which has contributed to the generation of the national imagined communities in terms of Benedict Anderson [1]. They are not an ‘objective’ structural feature but more a perspective of individuals and groups to observe, understand and reflect themselves in a social context. The issue is particularly relevant in the turbulent times of globalisation, mass migrations, intercultural clashes and their hardly predictable effects well illustrated by the recent refugee crisis in Europe.National identities are clearly related to modernity, which has contributed to the generation of the national imagined communities in terms of Benedict Anderson [1]. They are not an ‘objective’ structural feature but more a perspective of individuals and groups to observe, understand and reflect themselves in a social context. The issue is particularly relevant in the turbulent times of globalisation, mass migrations, intercultural clashes and their hardly predictable effects well illustrated by the recent refugee crisis in Europe.This semantic shift corresponded well to the break with the semantic of the pre-modern stratified society—replacing the old hierarchically based semantic distinction between the feudal aristocracy and its subjects with the distinction between different ‘peoples’ thus developing their particular (national) identities. However, although national identities have initially clearly corresponded to the origins of modernity, the relationship between the two phenomena is more complicated, since all kinds of national identity are not necessary compatible with the further development of modern society. In general, modernity is clearly related to the concepts of inclusion. This implies that individuals are supposed to be included in all functional subsystems of the society [5,6], which is also well reflected in the Enlightenment based values of freedom and equality—as clear guiding principles of modern societies [7].On the other hand, the national semantics seems to be inevitably different. Initially, a person has been supposed to belong fully to a single nation implying certain exclusivity both at the social and at the personal side. The semantics defining national belonging in terms of “insiders” and “outsiders” includes different “ethnic philosophies” [8], which condition the two broad principal models of the nation state: the French universalist and the German referring to a cultural particularity [9]. Resembling those conceptions, Smith [10] defines the civic-territorial and the ethnic-genealogical model of the nation. It may be argued that the differences between the models may be understood as competing semantics.While the nation based divisions are communicated within the social system and self-described in terms of national identity semantics, it should be noted that the self-description of individuals also includes a semantic of their own (national) identities and belongings and of attributing them to others [11]. The transformations in the semantics referring to national belongings have thus occurred both at the individual and the social level. With the increasing complexity of the social systems [12], individuals have been entering a variety of subsystems and become confronted with the multiple meanings produced by communication, which have had certain impact on individuals’ cognition. Social systems and individuals should be seen as two emergent units having their own causal powers [13]. Mutual influences are restricted to the structural level of both systems, which determines the reproduction, meaning that “both types of systems are structurally adapted to each other in a way which allows for mutual irritation” [14]. In that regard, the important role is played by “trigger-causality” [6,14], meaning that individual is a trigger to social systems, whose structures respond in their own specific way of reproduction, and vice versa. Social system is a trigger to cognition of individual, who responds in her or his own specific way. Individuals’ social embeddedness reflects the variety of social environments, which have a potential to ‘trigger’ them. The link between social forces (expressing all possible triggers of environments provided by social system) and individuals is made by confrontation between communication, which is an emergent property of social systems, and thoughts, cognition of individuals.In order to cope with growing modernisation, the reduction of meanings in self-description of social systems has brought to communication, which supports certain transformations in national belongings. Resulting from the interplay between emergent structural properties of social systems and individuals, one can notice the semantics of individuals’ self-describing in terms of national belongings have responded to those structural transformations. Individuals have begun to express different forms of national identity but they are not all equally compatible with further modernisation.

## Transforming National Belongings: The Framework of This Study

The result of structural coupling between cultural information instigated by cognitive frames, determined by social structure, and individuals’ cognition can be understood in terms of cultural memory. The latter is characterised by its distance from the everyday and preserves the store of knowledge of unity and peculiarity of one group [15]. National identities reflect the semantics of self-describing referring to a cultural memory, which is seen as a particular form of mediated action of human agents who make use of cultural repertoires or so-called cultural tools. The availability of cultural tool kits depends on agents’ position in social structure specific to a certain time and place [16]. Even within the classical social order, the distinction in the semantics of identity can be observed: between the ascribed and the voluntarist criteria of national belonging, where—as we shall hypothesise—only the relative inclusiveness of the latter may be compatible with development in terms of modernisation (since the semantics related to modernity also implies inclusiveness). The classical social order may thus be seen as early modernisation since the emerging semantics of national identity already reflects a shift away from the pre-modern stratified social system. Regarding the classical social order, we thus hypothesise that the ascribed national identities are the most typical for the least developed societies, lagging behind in terms of modernisation (Hypothesis 1), while the voluntarist national identities are compatible—though not necessarily correlated with modern development (Hypothesis 2). On the other hand, in an emerging social order of the more developed, so called second modernity [17], manifested through further human and social development, the semantics or cultural information has become multiple, often contested and ambiguous. The increasing number of variations of the social systems provides a new environment for the individuals, in which meanings become routinely questioned and ‘irritate’ the individuals’ noise. Particular narratives and images are not just reproduced and reframed [18], but often put into question through new cultural information playing a protagonist role in the construction of identities, which can be individual or collective. Changed cultural information provides a construction of new memories, which respond to contemporary cultural complexities [18]. The transformed processes of memory transmission can be understood by using Benjamin’s distinction between ‘transmitted’ and ‘acquired’ memory. The construction of new memories refers to acquired memory denoting what happens to individual alone, and it has becoming more and more significant in human lives [19]. Excessive number of choices and high risk-taking resulting from increasing complexity of society demand rapid and efficient adaptation of individuals to their environment. Self-description in terms of contemporary identifications is a means of reducing instability and hyper complexity of meanings. Globalisation processes have changed the material and imaginary relationship toward space generating the self-descriptions of imagined communities reaching beyond Anderson’s conceptualisation of nation state [1]. The classical meanings of ‘the nation’ can no longer be the principal site of frame of cultural memory because the new acquired memories refer more to post-national cultural constellations [18]. In the emerging social order, one can thus construct instrumental transnational or even supranational identifications, which co-exist with the national ones. The semantics referring to ascribed elements of national belongings is of different nature as in the classical social order and support the efficient adaptation of individuals to the environment [20–22]. Common myths, origin and the idea of the territorial homeland are for instance a powerful source instrumentalised by transnational diasporic communities. Unfortunately, the available survey data does not allow us to test the relationship between this type of identity and modernisation, which thus remains a challenge for further research.

A step further is taken by supranational instrumental identifications that are based on pragmatic choices and fall fully in line with the decreasing relevance of the national borders and the supranational nature of the functional subsystems [20,23], well-illustrated by the case of the European Union. We hypothesise that these types of identity semantics are not only compatible but even positively correlated to the further development of modern society (Hypothesis 3). The typology of identity constructions, their relationship with the development of modernity and the specification of our hypotheses are presented in Table 1.

**Table 1:** A typology of identity constructions.

	Types of identity semantics	Hypotheses: compatibility / relationship with development
**Classical social order**	Classical ascribed national identity: firm national identity, transferred inter-generationally, e.g. ethno-nationalism.	Hypothesis 1: low compatibility—expecting significant negative statistical relationship.
	Classical voluntarist national identity: Firm civic identity, strong sense of inclusive citizenship, e.g. U.S. or French patriotism.	Hypothesis 2: medium compatibility—expecting no significant statistical relationship between the phenomena.
**Emerging social order**	Instrumental transnational identifications: Instrumentalisation of national traditions, re-inventing or searching for national (ethnic) roots, e.g. among the next generations of immigrants.	Not tested due to unavailable survey data. Medium compatibility may be expected.
	Instrumental supranational identifications: Instrumental identification based on pragmatic choices, e.g. EU citizenship.	Hypothesis 3: high compatibility—expecting significant positive statistical relationship.

### Analysis: Indicators of national identity

The link between the individuals and social systems and their self-descriptions in terms of identity, briefly explained in sections 2 and 3, is required not only for theoretical clarification but also for the operationalisation needed for empirical testing of our hypotheses. This is at least because the social semantics of national identity is measured in social surveys at the level of individual responses. A potentially possible replacement of individual level analysis with (e. g. national) statistical aggregates would simplify the analysis but it would also imply a significant (and unnecessary) loss of information, since different individuals may participate in social systems in various ways and may thus also reflect the semantic of national identity in a broad variety of ways. We intend to test, through correlation and hierarchical regression methods [24], whether modern development also generates different types of national identities. The dataset from the International Social Survey Programme (ISSP) from 2003, is a part of a continuing annual programme of cross-national surveys covering important topics for social science research [25]. It contains several variables regarding the criteria of national identity, i.e. the criteria considered as important by the respondents to define a person to belong to a certain nation. They are listed in the first column of as Table 2. Confirming strong correlations between some of them we performed Principal Component Analysis. Results showed that by the two extracted components 58% of total variance was explained. Based on this, two separate components were detected in an almost identical manner [26] on the previous ISSP dataset from 1995 [27]. In line with Smith and Jones [26] and our own definitions explained in section 3 (see Table 1; the rows regarding Classical Social Order), we interpreted these two components as (1) ascribed criteria and (2) voluntarist criteria. As presented in Table 2, the first (ascribed) component has high loadings of being born in the country (variable Born in our analysis), living there for most of one’s life (variable MostLife) and belonging to the dominant religion (variable Religion). The second (voluntarist) component, on the other hand, has high loadings for respecting the country’s political institutions and laws (Institutions), the feeling of country’s nationality (Feel) and being able to speak the language (Language). Citizenship has a more ambivalent meaning, which is hardly surprising because of its combined nature: being ‘a civic’ element on the one hand but also strongly conditioned by birth and long-term residence in a given country (see Table 2).

**Table 2:** Principal Component Analysis—Rotated Component Matrix of national belonging criteria. Source: ISSP Research Group 2003 [25]; own calculations.

	Component	
	1 ‘ascribed criteria’	2 ‘voluntarist criteria’
Important: to have been born in [Country]	**0,838**	0,102
Important: To have [Country Nationality] citizenship	**0,641**	0,422
Important: To have lived in [Country] for most of one's life	**0,752**	0,277
Important: To be able to speak [Country language]	0,283	**0,632**
Important: To be a [religion]	**0,652**	0,057
Important: To respect [Country Nationality] political institutions and laws	-0,057	**0,840**
Important: To feel [Country Nationality]	0,353	**0,635**

However, the operationalization of the national identity and its intensity is a more complicated issue than implied by Smith and Jones [26]. What the variables presented in Table 2 actually measure is the significance attributed to various criteria applied when considering others as members or non-members of a particular imagined (national) community. On the other hand, these elements as such tell nothing about the intensity of individuals’ own feelings of national identity and the relevance of national identity for them. For instance, an individual who claims that that birth and residence are important for national identity does not necessarily believe that national identity as such is important.

A proper indicator of intensity of national feelings may be found in the same dataset under the question on ‘how close’ one feels to one’s country (variable CloseCountry). We should thus combine the question of intensity of national belonging (CloseCountry) with the set of questions regarding the criteria of national belonging (as presented in Table 2) in order to establish the indices of ascribed and voluntaristic national identity. The combination is based on the multiplication of each criterion with the general intensity measured by the CloseCountry variable and summing them up for the overall assessment. We assume that the variables distributed into the two categories established through the principal component analysis presented in Table 2 all equally contribute to the each of the two types of national identity. Feelings of different individuals may involve different weights but there is no persuasive theoretical or empirical argument that would support a single variable being more important in general than any other. Consequently, we put equal weights to all variables of the national identity types (or criteria) since different weights would be arbitrary. The citizenship criterion was deliberately left from the index formation since it is not—as we have noted above—clearly positioned into either the ascribed or the voluntarist dimension. The criteria for national belonging measured originally at the Likert scale ranging from 1 to 4 were for the purpose of our analysis recoded into: 2 for seeing a given feature as ‘very important’, 1 for seeing it as ‘important’, 0 ‘for seeing it as ‘not very important’ and -1 for seeing it as ‘not important at all’. This implies that a given criterion can be strongly emphasised (value 2), seen as relevant (value 1), not seen as relevant (value 0) or even seen as something that should not be considered at all (value -1). One’s closeness to one’s country measured originally at the Likert scale ranging from 1 to 4, was recoded into: 2 (‘very close’), 1 (‘close’) and 0 for either ‘not very close’ or ‘not close at all’. This implies that national identity in relation to the country where a person lives may be very intensive (value 2), existing but not very intensive (value 1) or non-existent (value 0). For the purpose of our analysis we have deliberately ignored the difference between ‘not very close’ and ‘not close at all’ since it is not our intention to measure ‘negative’ national identities in terms of rejecting one’s country—such identities are only seen here as the lack of national identity. Based on these considerations and values, the Ascribed National Identity Index (*Ascribed*) and Voluntarist National Identity Index (*Voluntarist*) were calculated as follows:

Ascribed=CloseCountry×(Born+MostLife+Religion) (1)

Voluntarist=CloseCountry×(Language+Institutions+Feel) (2)

Finally, in order to assess the presence of supranational identities that may be typical for the emergent order described in Section 3 (see Table 1, the row on ‘supranational identifications’), we took the distinction from Eurobarometer 2012 dataset [28] between the persons who feel themselves as European citizens and the rest as an indicator of supranational identity (EUCIT). Thus we created a dichotomous variable with the value of 1 for those who considered themselves as the EU citizens and 0 for those who did not.

## Results

**Fig 1:**
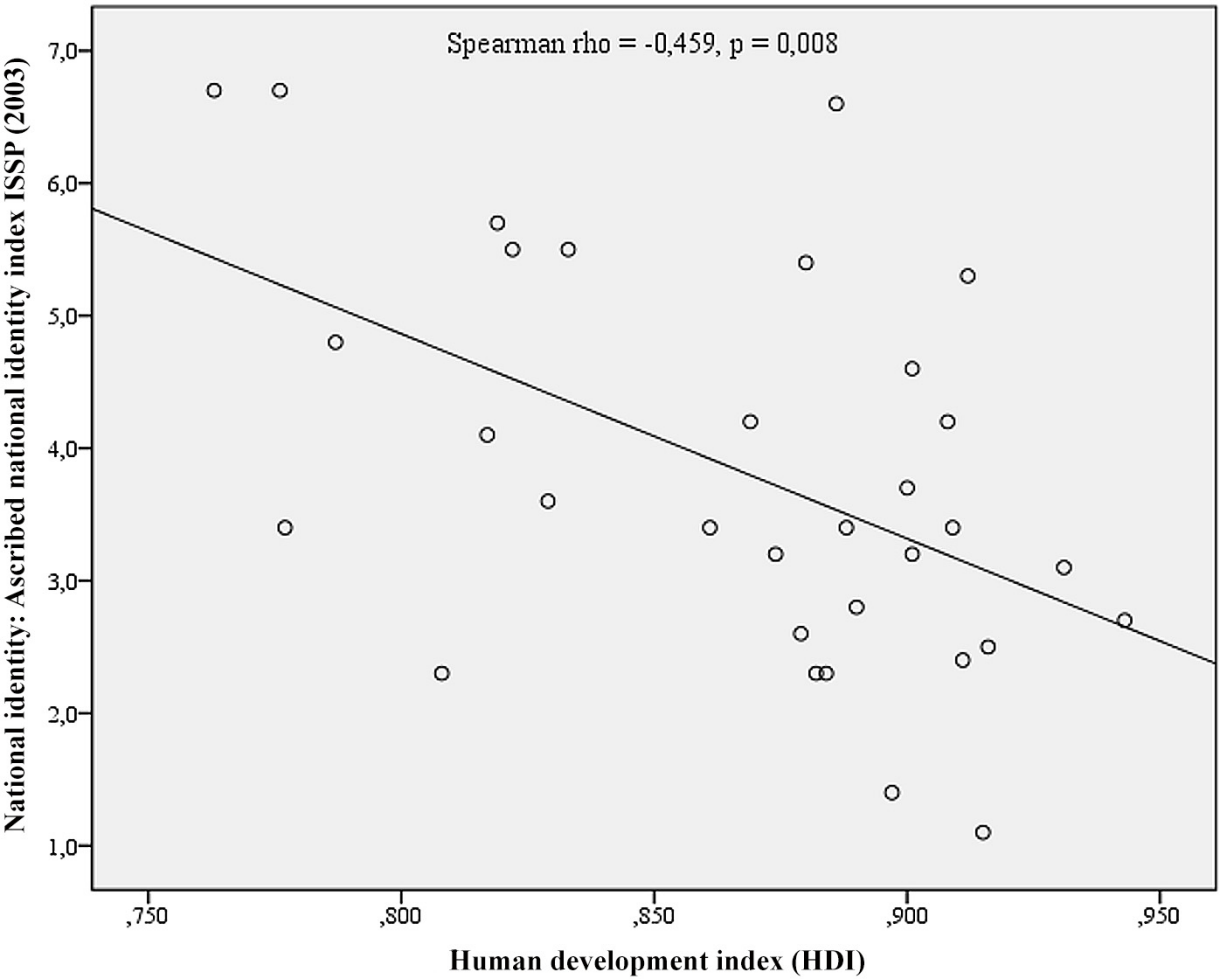
Human Development and Ascribed National Identity at the national levels. The scatterplot in Fig 1 clearly demonstrates the decreasing ascribed national identity with the higher levels of development.

**Fig 2:**
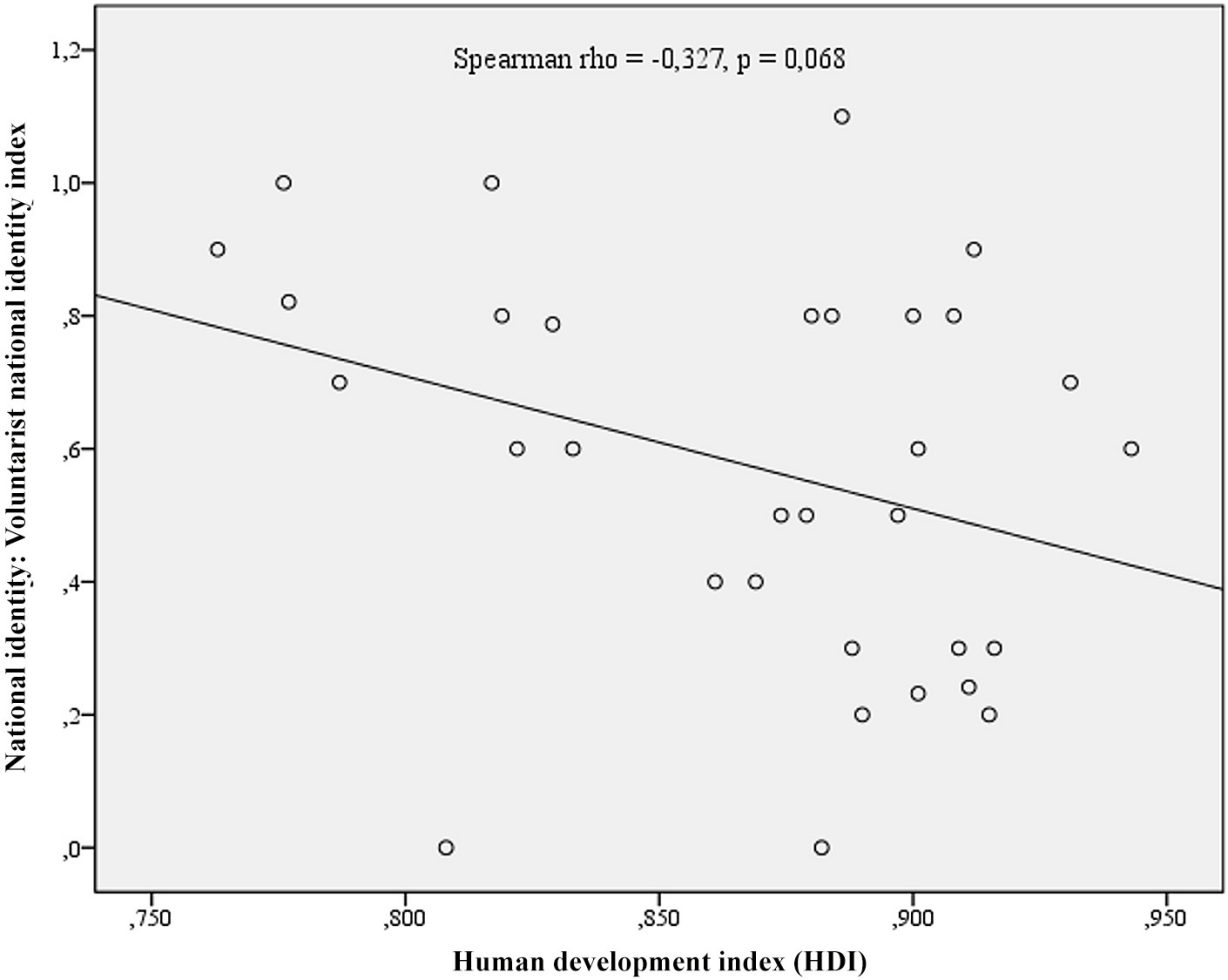
Human Development and Voluntarist National Identity at the national levels. Fig 2, on the other hand, indicates inevitably high levels of voluntarist identity at lower levels of development, while the results for highly developed nations may vary from quite low to quite high levels of voluntarist identity.

**Fig 3:**
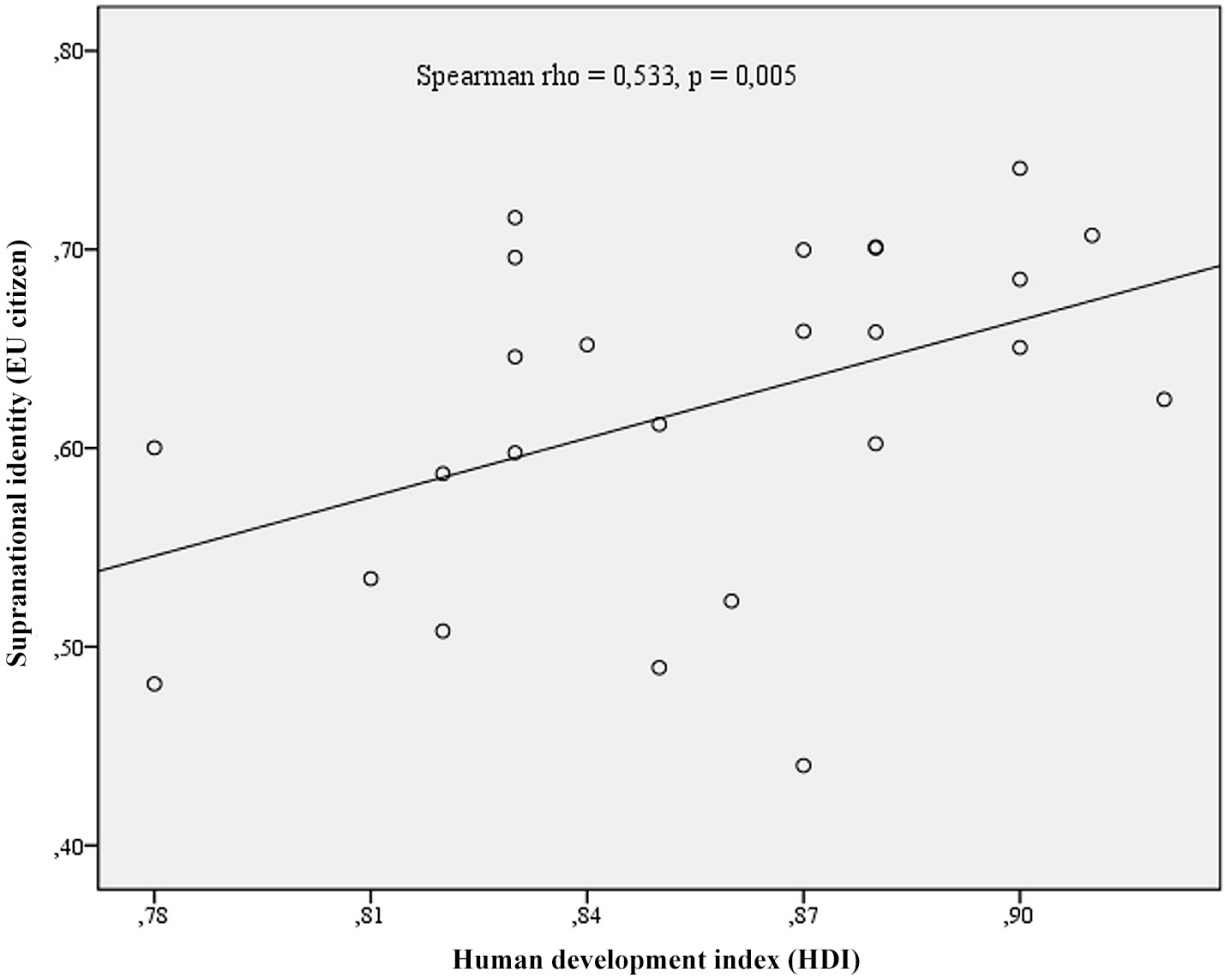
Human Development and the feeling of EU citizenship at the national levels. Regarding the supranational instrumental identifications of the emerging social order, EU countries with higher HDI are more likely to demonstrate higher levels of supranational identities in terms of feelings of European citizenship. The relationship is presented as a scatterplot in Fig 3.

The relationships between development and identity are presented in Fig 1 for Ascribed National Identity and Fig 2 for Voluntarist National identity. The scatterplot in Fig 1 clearly demonstrates the decreasing ascribed national identity with the higher levels of development. Fig 2, on the other hand, indicates inevitably high levels of voluntarist identity at lower levels of development, while the results for highly developed nations may vary from quite low to quite high levels of voluntarist identity. In other words, with modern development, ascribed national identity decreases (making it incompatible with modern development), while the voluntarist may either decrease or not (making it compatible with modern development since the negative statistical correlation is only caused by the consistently high levels of voluntarist national identities at the low levels of human development). Regarding the supranational instrumental identifications of the emerging social order, EU countries with higher HDI are more likely to demonstrate higher levels of supranational identities in terms of feelings of European citizenship [29]. The relationship is presented as a scatterplot in Fig 3.

Although this speaks in favour of our hypotheses, we should perform the analyses at the individual level as well. Controlling for the individual socio demographic characteristics, which may affect one’s structural position and thus the perception of national identity [10,26], is required. We should also consider a potential impact of one’s immigrant origins that may generate competing trans-national identifications and contribute to declining *classical* national identity. For these purposes we use a two-level hierarchical linear regression HML2 equation available in HLM7 software with HDI as the second level independent variable as stated bellow:

$$ACRIB_{ij}=\gamma_{00}+\gamma_{01}\times HDI_{j}+\gamma_{10}\times AGE_{ij}+\gamma_{20}\times EDUCYRS_{ij}+\gamma_{30}\times FEMALE_{ij}+\gamma_{40}\times PNOTC_{ij}+u_{0j}+r_{ij}$$ (3)

where ASCRIB stands for Ascribed National Identity Index, HDI for Human Development Index, AGE for individual’s age, EDUCYRS for the number of years of completed education, FEMALE for being a female, PNOTC for respondent’s parents not being citizens of her or his country (implying a migrant origin), level 2 error term *u*_0*j*_ and error term *r*_*ij*_.The model in Fig 2 is based on the hypothesis 1 implying that HDI within a given national context will independently decrease the levels of ascribed national identity (as the dependent variable in the model) while controlling for the individuals’ features including age, education, gender and immigrant origins (see Table 3).

**Table 3:** Final estimation of two levels hierarchical regression with Ascribed National Identity Index as outcome variable on individual level.

Fixed Effect	Coefficient	Standard error	*t*-ratio	Approx. *d*.*f*.	*p*-value
**For INTRCPT1, *β***_***0***_					
**INTRCPT2, *γ***_***00***_	3,603	0,236	15,279	30	<0,001
**HDI, *γ***_***01***_	-14,705	4,871	-3,019	30	0,005
**For AGE slope, *β***_***1***_					
**INTRCPT2, *γ***_***10***_	0,054	0,001	51,000	42274	<0,001
**For EDUCYRS slope, *β***_***2***_					
**INTRCPT2, *γ***_***20***_	-0,005	0,001	-6,078	42274	<0,001
**For FEMALE slope, *β***_***3***_					
**INTRCPT2, *γ***_***30***_	0,227	0,037	6,165	42274	<0,001
**For PNOTC slope, *β***_***4***_					
**INTRCPT2, *γ***_***40***_	-2,066	0,077	-26,760	42274	<0,001

**Fig 4:**
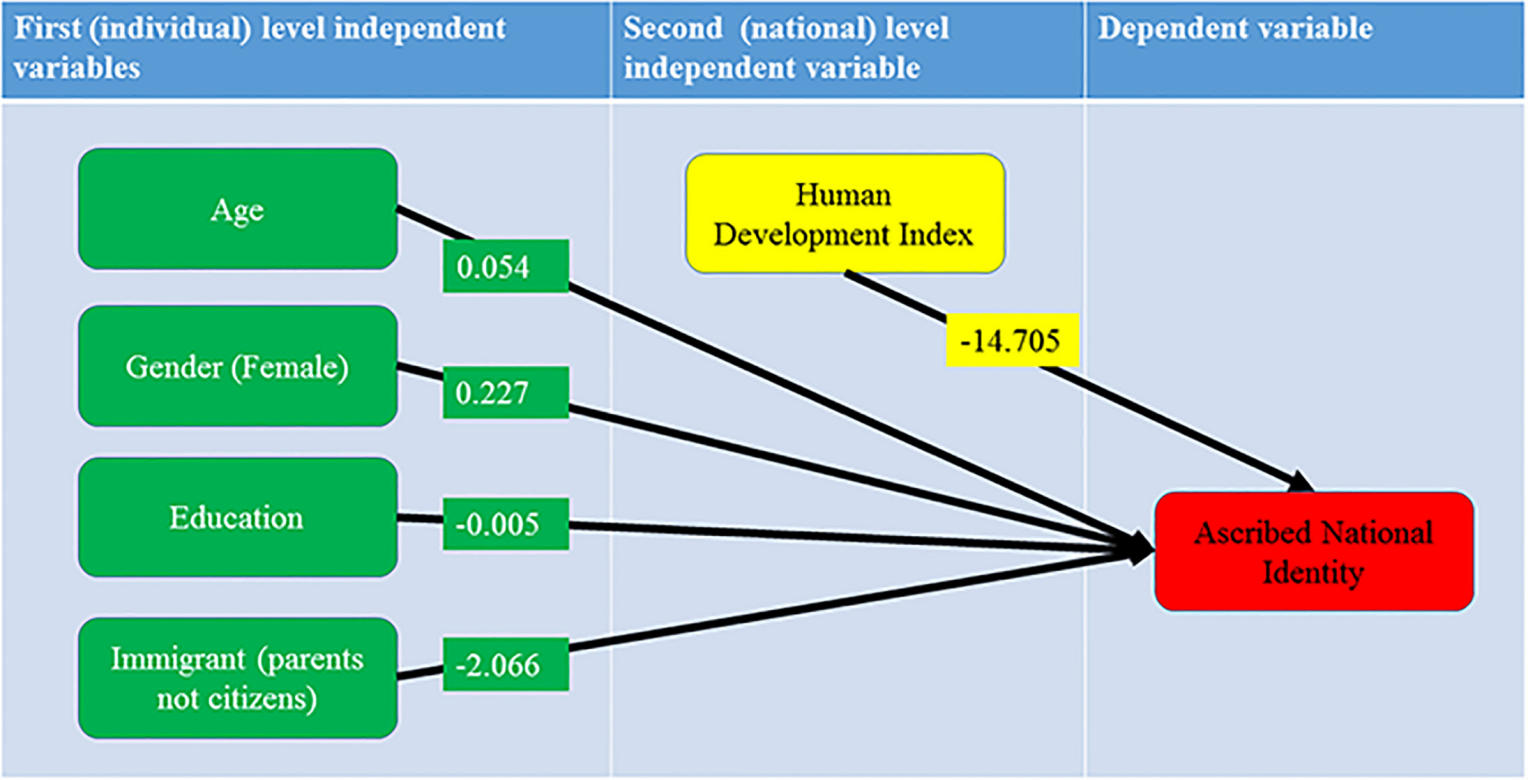
The hierarchical regression model for the Ascribed National Identity Index with statistically significant first level regression coefficients. Fig 4, confirm the significance of the first level regression coefficients for all variables included in the model and significant impact of HDI as the second level variable. HDI with the negative and significant effect confirms that the macro level HDI variable tends to contribute to the decreasing levels of ascribed national identity.

The results in Table 3, presented graphically in Fig 4, confirm the significance of the first level regression coefficients for all variables included in the model and significant impact of HDI as the second level variable. HDI with the negative and significant effect confirms that the macro level HDI variable tends to contribute to the decreasing levels of ascribed national identity. Moreover, at the individual level the dependent variable is significantly affected by the immigrant origins of a respondent. It is very likely that an immigrant position generates competing instrumental cross-national identities instead of classical ascribed national identities (see Table 1).

A similar model has been constructed for voluntaristic national identity as well, namely:

$$VOLUNT_{ij}=\gamma_{00}+\gamma_{01}\times HDI_{j}+\gamma_{10}\times AGE_{ij}+\gamma_{20}\times EDUCYRS_{ij}+\gamma_{30}\times FEMALE_{ij}+\gamma_{40}\times PNOTC_{ij}+u_{0j}+r_{ij}$$ (4)

where *VOLUNT* stands for our Voluntarist National Identity Index.

**Fig 5:**
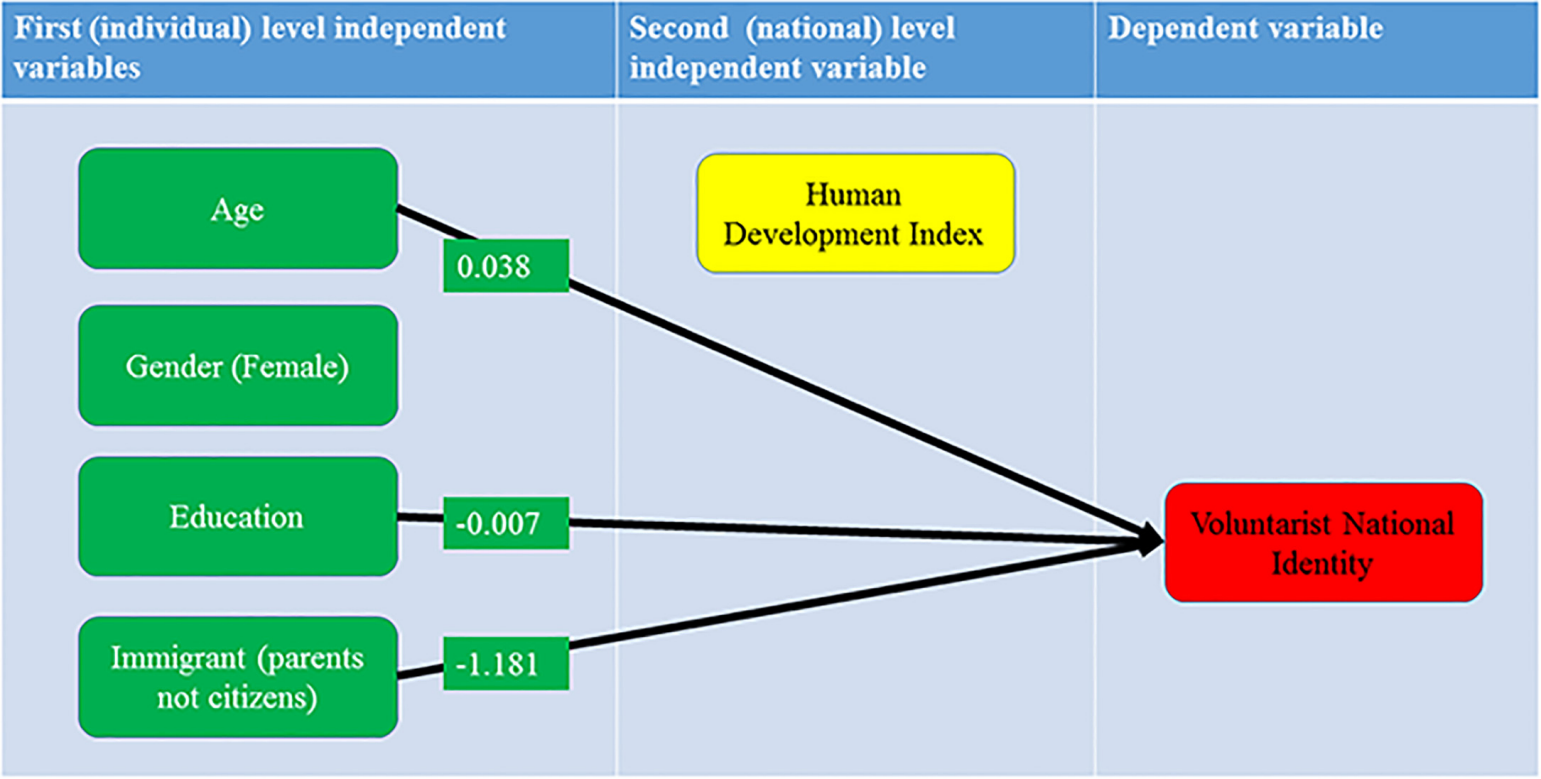
The hierarchical regression model for the Voluntarist National Identity Index with statistically significant first level regression coefficients. Fig 5 confirm hypothesis 2 since they demonstrate no relevant impact of HDI on the voluntarist national identity even when individual level variables (age, education, gender and immigrant origins) are also controlled for.

The results presented in Table 4, presented graphically in Fig 5, confirm hypothesis 2 since they demonstrate no relevant impact of HDI on the voluntarist national identity even when individual level variables (age, education, gender and immigrant origins) are also controlled for.

**Table 4:** Final estimation of two levels hierarchical regression with Voluntarist National Identity Index as outcome variable on individual level.

Fixed Effect	Coefficient	Standard error	*t*-ratio	Approx. *d*.*f*.	*p*-value
**For INTRCPT1, *β***_***0***_					
**INTRCPT2, *γ***_***00***_	5,808	0,202	28,730	30	<0,001
**HDI, *γ***_***01***_	-4,056	4,604	-0,881	30	0,385
**For AGE slope, *β***_***1***_					
**INTRCPT2, *γ***_***10***_	0,038	0,003	14,379	42274	<0,001
**For EDUCYRS slope, *β***_***2***_					
**INTRCPT2, *γ***_***20***_	-0,007	0,001	-4,807	42274	<0,001
**For FEMALE slope, *β***_***3***_					
**INTRCPT2, *γ***_***30***_	0,057	0,065	0,877	42274	0,380
**For PNOTC slope, *β***_***4***_					
**INTRCPT2, *γ***_***40***_	-1,181	0,267	-4,423	42274	<0,001

Finally, the hypothesis 3 was tested using a nonlinear multilevel logistic regression model [30]:

$$EUCIT_{ij}=\gamma_{00}+\gamma_{01}\times HDI_{j}+\gamma_{10}\times AGE_{ij}+\gamma_{20}\times EDUCATER_{ij}+\gamma_{30}\times FEMALE_{ij}+u_{0j}$$ (5)

where *EUCIT* stands for the indicator of supranational identity, *AGE* for individualpranatio*EDUCYRS* for the number of years of completed education, *FEMALE* for being a female and level 2 error term *u*_0*j*_.

**Fig 6:**
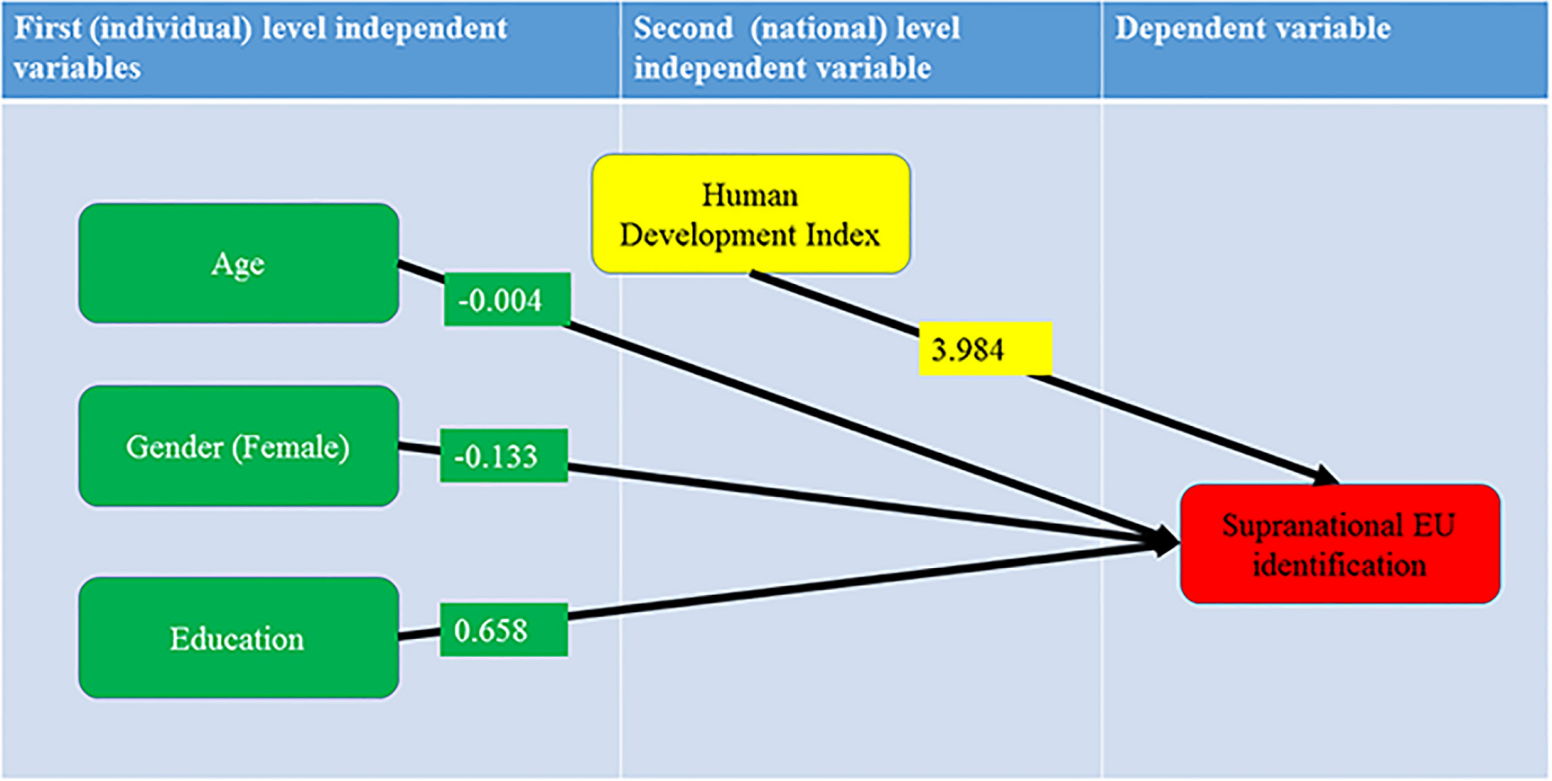
The hierarchical regression model for the EU supranational identification with statistically significant first and second level regression coefficients. While controlling for the (significant) effects of age, gender and education, the independent positive effect of HDI on supranational identity was confirmed as showed in Fig 6.

While controlling for the (significant) effects of age, gender and education, the independent positive effect of HDI on supranational identity was confirmed as showed in Table 5. It may thus be claimed that HDI contributes to the semantics of supranational identities (see Fig 6), such as the European one.

**Table 5:** Final estimation of two levels Bernoulli distributed hierarchical regression with indicator of supranational EU identity as outcome variable on individual level.

Fixed Effect	Coefficient	Standard error	*t*-ratio	Approx. *d*.*f*.	*p*-value
**For INTRCPT1, *β***_***0***_					
**INTRCPT2, *γ***_***00***_	0,519	0,069	7,536	24	<0,001
**HDI, *γ***_***01***_	3,984	1,526	2,610	24	0,015
**For AGE slope, *β***_***1***_					
**INTRCPT2, *γ***_***10***_	-0,004	0,002	-1,663	22821	0,096
**For EDUCATER slope, *β***_***2***_					
**INTRCPT2, *γ***_***20***_	0,658	0,045	14,742	22821	<0,001
**For FEMALE slope, *β***_***3***_					
**INTRCPT2, *γ***_***30***_	-0,133	0,028	-4,745	22821	<0,001

At the end of the modelling process, analysis of model fit and distributional assumptions of the models were computed on residual level– 1 and level– 2 files generated by HLM7 software. Residual analysis in examining the adequacy of the fitted models shows satisfactory results [31].

## Conclusions

National identity does not make nations more or less developed but the levels of national development in terms of material welfare, health and education are reflected in certain types of national identities. *We can thus confirm that a certain level of national development generates a particular type of national identity*.

Underdeveloped nations are thus significantly more likely on average to generate strong ascribed national identities, even when we control for the individual differences based on gender, age, education and immigrant origins. The lack of modernisation and its corresponding values of general inclusivity, openness, free choice, achievement based meritocracy, etc. would produce a comparatively closed and exclusivist type of identity based on birth, territory and religion.

Instrumental supranational identifications, on the other hand, will intensify with the higher levels of development. We have demonstrated this for the case of the European identity (in terms of EU citizenship): on average, the Europeans from the more developed member countries are more likely to indicate their instrumental supranational identification with the EU while controlling for the individual differences based on gender, age and education. This is especially notable with regard to the traditional Euro-sceptical attitudes in the U.K. (which is an actual exception from the rule as a clear outlier with lower EU identification than expected based on its level of development), Denmark and Sweden as some of the most developed European countries and the significant financial benefits received within the EU by the less developed member states. Our findings may be explained by the fact that instrumental supranational identifications presuppose high levels of modern development a confronting the people with new varieties of complex choices, including them in different and hardly predictable social realities—thus making the people more open for new uncertainties, more pragmatic and instrumental. The instrumental nature of EU supranational identifications reflects this.

Finally, firm voluntarist national identities tell one less about the overall levels of development. One can find them either in less developed societies, where they coexist with the ascribed ones, or in the most developed ones, coexisting with the instrumental supranational identifications. What makes them closer to the less modern societies may be the firm national belonging. However, its relative inclusiveness based on the civic instead of the ethnic-tribal criteria, makes them compatible with developed modern features as well.

Instrumental transnational identifications that may characterise the people of immigrant origins in particular remain unexplored in our research due to the lack of proper survey data. However, we have been able to find out in our regression models that being an immigrant (i.e. with parents not being citizens of the country) makes both voluntarist and oth voluntaristitizens of the countrysignificantly less intensive. This may speak in favour of the (theoretical) assumption that the immigrants would tend to develop instrumental transnational identifications instead of classical national identities. Nevertheless, the issue remains open for further systematic empirical testing.

## Supporting Information

S1 File Ascribed and Voluntarist National Identity on the individual level. (SAV)

S2 File Ascribed and Voluntarist National Identity on the country level. (SAV)

S3 File Supranational EU identity on the individual level. (SAV)

S4 File Supranational EU identity on the country level. (SAV)
